# Targeted mRNA demethylation in Arabidopsis using plant m6A editor

**DOI:** 10.1186/s13007-023-01053-7

**Published:** 2023-08-09

**Authors:** Ruiqiu Fang, Xiaolong Chen, Jie Shen, Bin Wang

**Affiliations:** 1 Institute of Maize and Featured Upland Crops, Zhejiang Academy of Agricultural Sciences, Dongyang, 322100 Zhejiang China; 2 Department of Life Sciences, Changzhi University, Changzhi, 046011 Shanxi China; 3 Institute of Vegetables, Zhejiang Academy of Agricultural Sciences, Hangzhou, 310021 Zhejiang China

**Keywords:** N6-methyladenosine, Plant m6A editors, Arabidopsis, dLwaCas13a, ALKBH5

## Abstract

**Background:**

N6-methyladenosine (m6A) is an important epigenetic modification involved in RNA stability and translation regulation. Manipulating the expression of RNA m6A methyltransferases or demethylases makes it difficult to study the effect of specific RNA methylation.

**Results:**

In this study, we report the development of Plant m6A Editors (PMEs) using dLwaCas13a (from *L. wadei*) and human m6A demethylase ALKBH5 catalytic domain. PMEs specifically demethylates m6A of targeted mRNAs (*WUS*, *STM*, *FT*, *SPL3* and *SPL9*) to increase mRNAs stability. In addition, we discovered that a double ribozyme system can significantly improve the efficiency of RNA editing.

**Conclusion:**

PMEs specifically demethylates m6A of targeted mRNAs to increase mRNAs stability, suggesting that this engineered tool is instrumental for biotechnological applications.

**Supplementary Information:**

The online version contains supplementary material available at 10.1186/s13007-023-01053-7.

## Background

RNA methylation regulates gene expression at the post transcriptional level and is an important epigenetic regulatory mode [1]. Over 200 types of post-transcriptional RNA modifications have been identified in plants. N6-methyladenosine (m6A) is the most common type of RNA methylation modification on higher biological mRNAs [2, 3]. m6A methylation is reversibly regulated by methyltransferase and demethylases complex [4]. These components of m6A modification complex are highly conserved across the plant kingdom [5]. m6A mark is involved in regulating mRNA processing, development, and stress response in plants [5]. m6A methylation appears to be a useful strategy to regulate gene expression, plant development and physiological processes [6].

Due to the lack of effective means to detect m6A methylation, the research on m6A methylation had long been stagnant. Thus far, studies aiming to manipulate RNA m6A methylation have relied on modulating the expression of RNA methyltransferases or demethylases [7], which has the shortcomings of affecting broad RNA methylation, making it difficult to study the effect of specific RNA methylation. Therefore, it is important to create tools in plants that allow the manipulation of RNA methylation in a more locus-specific manner.

CRISPR/Cas system is a powerful tool for understanding biological function and dynamic variations of nucleic acids [8]. Catalytically dead Cas9 (dCas9), retains site-specific binding but lacks DNA cutting activity. The nuclease-inactive DNA-targeting Cas9 (dCas9) fused with epigenetic regulatory enzymes can manipulate epigenetic properties at specific loci, including DNA methylation and histone methylation/acetylation status [9]. A protein family related to Cas9, the Cas13 protein family, was shown to natively target RNA [10]. Similar to Cas9, nuclease-inactive RNA-targeting Cas13 (dCas13) retains its crRNA-guided RNA binding ability [10–12]. In this study, we used catalytically dead LwaCas13a (from *L. wadei*) [10] and the human m6A demethylase ALKBH5 catalytic domain [13] to develop a plant m6A editors (PMEs) that target demethylation of specific mRNAs in plants. We further applied the PMEs system on endogenous mRNAs in Arabidopsis and successfully suppress target mRNAs degradation, suggesting that this engineered tool is instrumental for biotechnological applications.

## Results

**Fig. 1:**
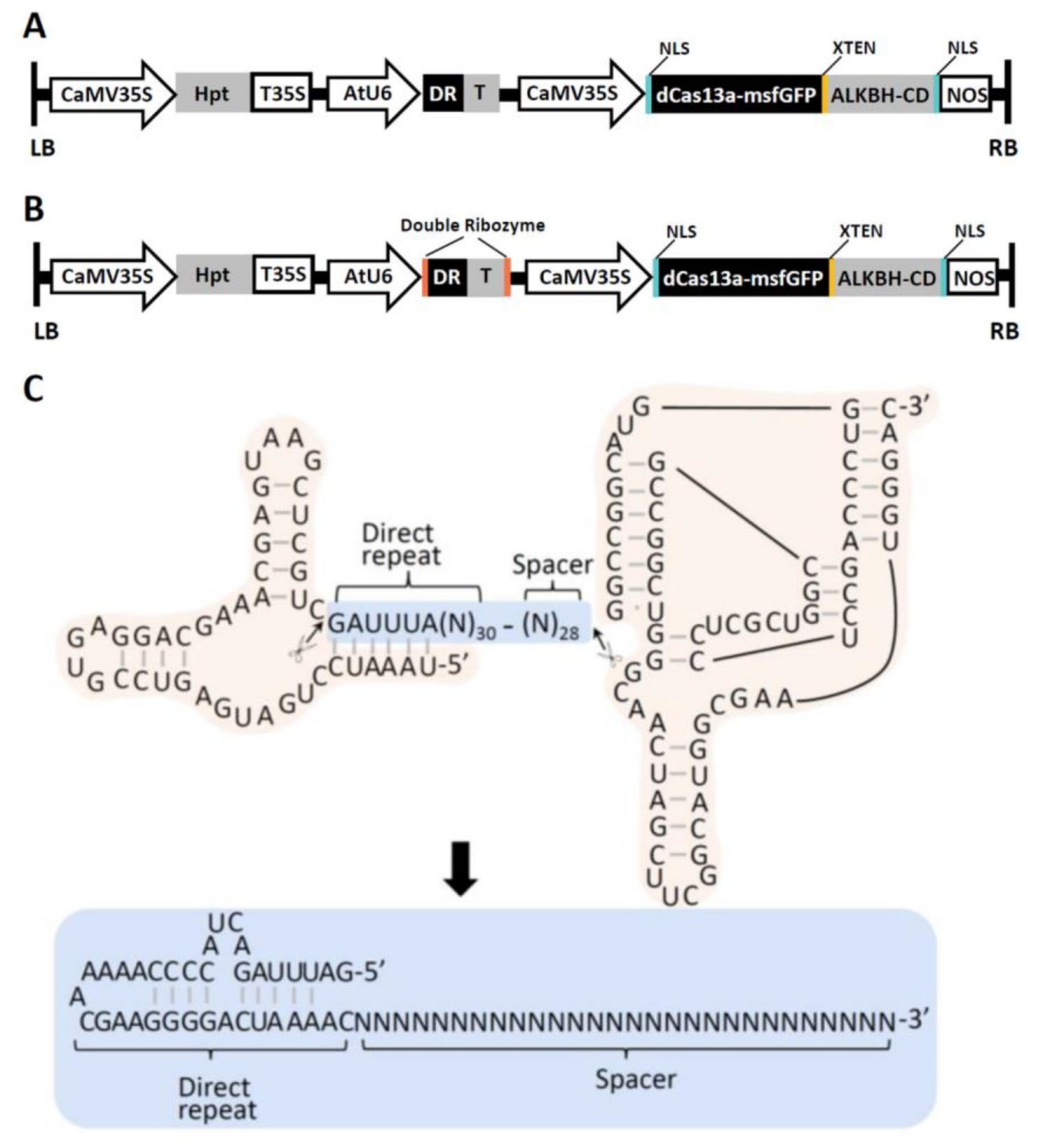
Schematic view of plant m6A editors (PMEs). **(A-B)** Schematic diagram of the construct for PMEs in Arabidopsis. ALKBH-CD represents human m6A demethylase ALKBH5 catalytic domain (66–292 aa); dLwaCas13a-msfGFP represents inactive dLwaCas13a (R474A and R1046A)-msfGFP; the XTEN linker separates dLwaCas13a-msfGFP and ALKBH-CD. dLwaCas13a-msfGFP-ALKBH-CD construct with two conserved nuclear localization signals (NLS) was driven by CMV 35 S promoter. RNA polymerase III promoter (AtU6) was employed to express crRNAs. For PME-WS-H and PME-FSS-H, double ribozyme system was used for crRNAs processing. DR indicates direct repeat and T indicates target for PMEs. **(C)** A self-cleaving double ribozyme system for precise processing of mature crRNAs. The upper graph is the predicted secondary structures o of the pre-crRNA, containing a Hammerhead ribozyme at the 5′-end (light yellow background), the sequence-specific crRNA portion in the middle (light blue background), and a HDV ribozyme at the 3′-end (light yellow background). The mature crRNA is released from the pre-crRNA through self-catalyzed processing. The mature crRNA contains direct repeat (universal for all crRNAs) and spacer (complementary to the target sequences)

To construct RNA editors, we synthesized human m6A demethylase ALKBH5 catalytic domain (66–292 aa) and fused it to the C-terminus of inactive dLwaCas13a (R474A and R1046A)-msfGFP structure using the unstructured 16-residue peptide XTEN as a linker [11, 12] (Fig. 1A; Additional file 1). Nuclear localization signal (NLS) peptides were added to the N-terminus of dLwaCas13a and C-terminus of ALKBH5. Catalytically inactive dLwaCas13a can be used as a programmable RNA binding protein. msfGFP was used to enhance the stability of the dLwaCas13a [10]. We expressed the dLwaCas13a-msfGFP-XTEN-ALKBH5 fusion sequence under the CMV promoter and the CRISPR RNA (crRNA) sequence under the Arabidopsis RNA polymerase III promoter AtU6 (Fig. 1A). The sequences of the crRNA might be highly conserved and important for Cas13a activity [10]. 3’ terminal poly U sequences present in RNA polymerase III-transcribed crRNAs are immediately adjacent to protospacer sequences involved in RNA recognition [14]. To meet the sequence specificity, we also used a double ribozyme system that precisely processes the crRNAs (Fig. 1A-B) [15]. Double ribozyme system contained a hammerhead (HH) type ribozyme [16] at the 5’-end, a crRNA, and a hepatitis delta virus (HDV) ribozyme [17] at the 3’‐end (Fig. 1C). After the self‐cleavage at the predicated sites, the mature crRNA was released (Fig. 1C).

**Fig. 2:**
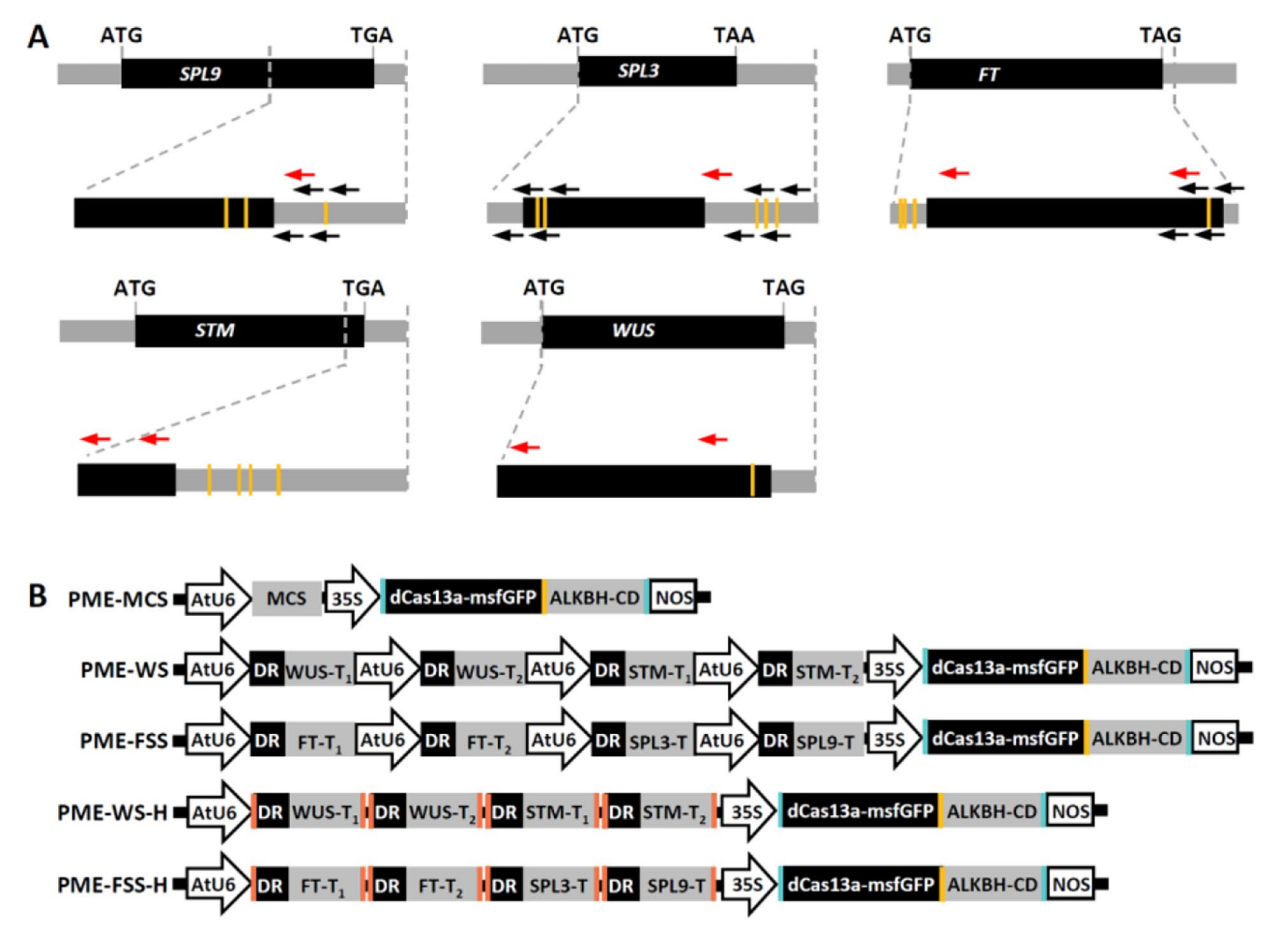
Design and construction of PMEs inducing targeted demethylation of *SPL9*, *SPL3*, *FT*, *WUS*, and *STM *mRNAs. **(A)** Schematic representation of positions of the m6A sites and regions targeted by crRNAs. The CDS and UTR are represented by black and gray boxes. Yellow bars represent the m6A sites. Red arrow indicates the crRNA. The crRNA was designed to target sequence near the m6A sites. Black arrows indicate the primers for SELECT-qPCR analysis. **(B)** Schematic illustration of PME-MCS, PME-WS, PME-FSS, PME-WS-H, and PME-FSS-H.

To test the PMEs system, we chose *WUS*, *STM*, *FT*, *SPL3*, and *SPL9* as the target genes (Fig. 2A; Additional file 2). Several m6A sites have been identified in the transcript of the five genes [7, 18]. *FT*, *SPL3*, and *SPL9* are key activator of flowering [7]. m6A methylation of *FT*, *SPL3*, and *SPL9* mRNAs affects floral transition. *STM* and *WUS* are two key shoot apical meristem (SAM) regulators [18]. m6A methylation on *STM* and *WUS* determines shoot stem cell fate in plants. According to Shen et al. (2016) and Duan et al. (2017), crRNAs sites were designed near the m6A sites of the five genes [7, 18] (Fig. 2A). Studies suggested that the secondary structure of crRNA are critical for the editing efficiency of Cas13a/crRNA [10, 19, 20]. Therefore, selection of target sequences should avoid disrupting the secondary structure of crRNA. In this study, the guide sequences targeting the m6A sites of the chosen genes were predicted with the software RNAfold (http://rna.tbi.univie.ac.at/cgi-bin/RNAWebSuite/RNAfold.cgi) (Additional file 3). In addition, we used two classes of construct for expression of crRNAs. Four units of crRNAs expression cassettes under four AtU6 promoters were ligated in tandem (PME-WS and PME-FSS), and four units of crRNA ribozyme cassettes under one AtU6 promoter were ligated in tandem (PME-WS-H and PME-FSS-H) (Fig. 2B). The four constructs together with control vector (PME-MCS) were transformed into Arabidopsis (Col-0) via *Agrobacterium Tumefaciens*-mediated transformation. The T_1_ transgenic plants were confirmed by PCR analysis of genomic DNA (Additional file 4). qPCR analysis showed the dLwaCas13a-msfGFP-XTEN-ALKBH5 was expressed in different transgenic lines (Additional file 5). To exclude the effect of transgene on endogenous gene expression, we analyzed the expression levels of orthologs of *ALKBH5* in transgenic plants. There are five potential orthologs of human *ALKBH5* encoded in the Arabidopsis genome: *ALKBH9A*, *ALKBH9B*, *ALKBH9C*, *ALKBH10A*, and *ALKBH10B* [7]. We analyzed the expression of the five genes in WT, PME-MCS #4, and PME-MCS #5, and found that there were no obvious expression changes in the transgenic lines (Additional file 6).

**Fig. 3:**
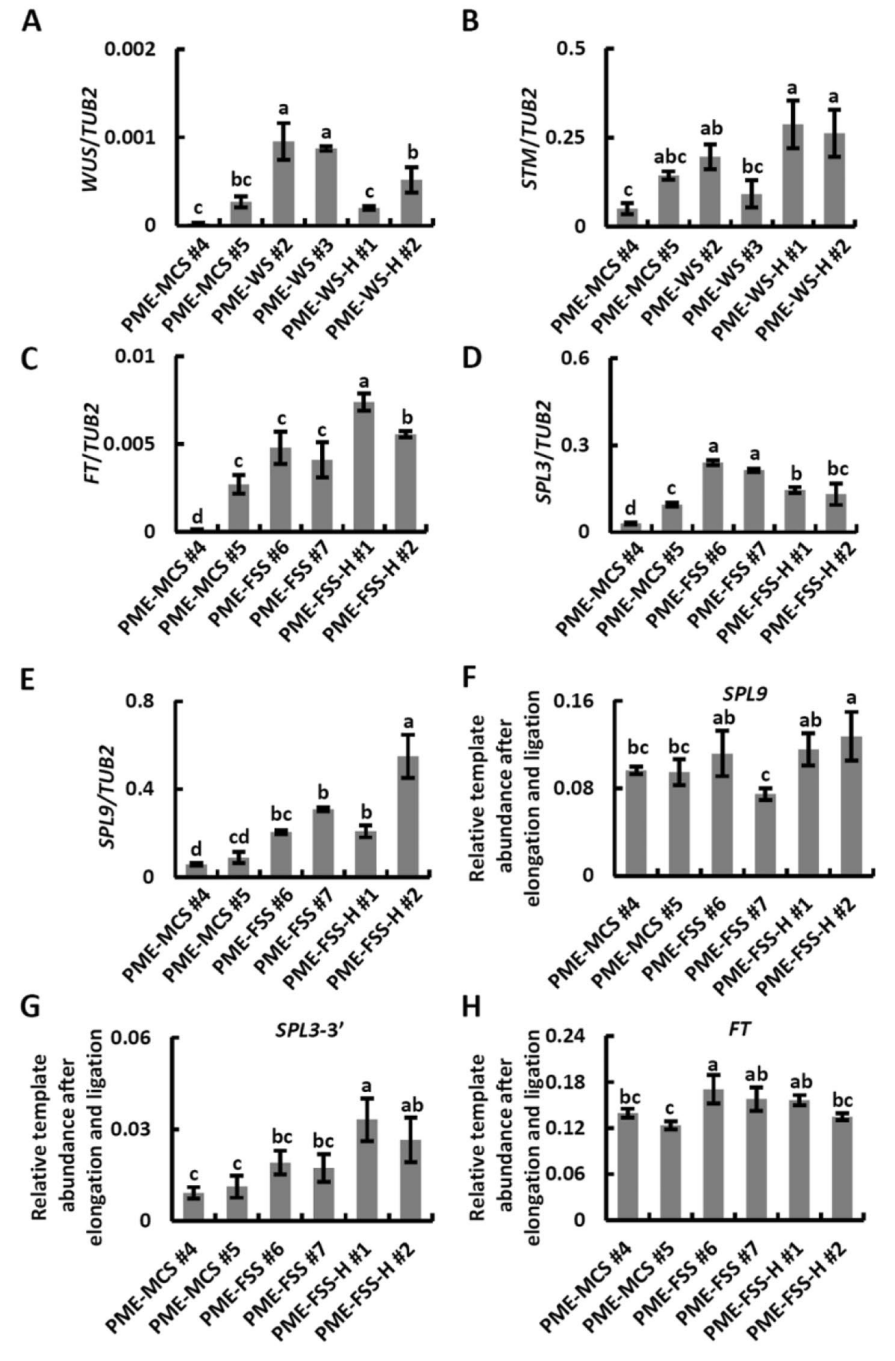
PMEs induces multiple demethylations of mRNAs.**(A-E)** qPCR analysis of the abundance of target mRNAs in T_3_ transgenic seedlings. *TUB2* was used as internal control. Error bars show SD (n = 3). Different letters at the top of each column indicate a significant difference at p < 0.05 determined by the Tukey test. **(F-H)** SELECT-qPCR analysis of the m6A level of target mRNAs in plants transfected with PME-MCS, PME-FSS, and PME-FSS-H, respectively. Error bars show SD (n = 3). Different letters at the top of each column indicate a significant difference at p < 0.05

We then verified the effect of PMEs on m6A modification of the selected genes in T_3_ Arabidopsis transgenic plants. It is generally believed that the m6A level is negatively correlated with target gene expression [3]. Therefore, we utilized qPCR assay to detect target gene expression. The results of qPCR showed that the four constructs (PME-WS, PME-FSS, PME-WS-H, and PME-FSS-H) targeting the selected genes can increase mRNA levels of the genes compared to the control vector (PME-MCS) (Fig. 3A-E). Notably, for constructs with the double ribozyme system, the mRNA levels of the selected genes were significantly increased. The results implied that PMEs might modify m6A level of target mRNAs. To determine the m6A level of target mRNAs, we performed SELECT-qPCR assay. SELECT-qPCR is a newly developed method for m6A level detection of target site with low cost and high efficiency. The m6A mark hinder the extension and ligation of template. Further qPCR analysis allows for quantification of relative template abundance after elongation and ligation [3]. In this study, SELECT-qPCR analysis revealed that PME-FSS and PME-FSS-H differentially decreased the methylation levels of *FT, SPL3, and SPL9* (Fig. 3F-H). In particular, the efficiency of crRNA targeting *SPL3* 3’UTR was higher than that of other crRNAs (Fig. 3F-H; Additional file 7A-D). Interestingly, PME-FSS-H with double ribozyme system showed higher modification efficiency, fold increasing relative to control. There are multiple m6A sites in *SPL3* mRNA, located near 5’ and 3’ UTR (Fig. 2A). To determine the modification efficiency of these sites by PMEs, we also detected change of m6A levels near *SPL3* 5’ UTR using SELECT-qPCR (Additional file 7E-H). Results showed that PME-FSS and PME-FSS-H could not effectively change the m6A level near *SPL3* 5’ UTR. Similar to Cas9-driven transcriptional activation and base editor [21, 22], PMEs system had limited editing scope. *FT*, *SPL3*, and *SPL9* are key genes regulating Arabidopsis flowering [7]. The expression level of the three genes showed a significant positive correlation with flowering in Arabidopsis. The number of rosette leaves at flowering was used to calculate the flowering period [23]. We counted the number of rosette leaves at flowering in T_3_ transgenic plants. Transgenic plants with PME-FSS or PME-FSS-H had fewer rosette leaves than plants with PME-MCS (Additional file 8 A-B). These results provided further confirmation of the early-flowering phenotype of PME-FSS and PME-FSS-H. Taken together, these data showed that targeted demethylation of functional gene transcripts can be efficiently generated using the PMEs system.

## Discussion

RNA methylation is an important mode of epigenetic regulation that regulates gene expression at the post-transcriptional level [1]. As the most common type of RNA methylation modification on higher biological mRNA, m6A methylation affects RNA stability and translational regulation in plants and animals [4]. Currently, studies involving m6A modification primarily rely on modulating the expression of RNA methyltransferases or demethylases [7], which has the shortcomings of affecting broad RNA methylation, making it difficult to study the effect of specific RNA methylation. In this study, we successfully applied plant m6A editors (PMEs) to perform targeted demethylation of specific mRNAs in Arabidopsis (Fig. 3A-H; Additional file 7). These observations combined suggested that this technology to a certain extent allows for targeted RNA demethylation, thus avoiding board epigenetic changes. Given the importance of m6A modification for eukaryotic mRNAs, we envisage that the PMEs system will be widely adopted to accelerate plant m6A methylation research.

In both mammalian and plant systems, the m6A methylation is crucial: knockdown of the gene *METTL3* encoding the m6A methyltransferase causes embryonic death in mice [24]. In plants, m6A is also required for normal development, and disruption of the m6A writer subunit leads to embryonic lethality in Arabidopsis [25–28] and early microspores degeneration of rice [29]. In addition, m6A is involved in various other physiological processes. The plant RNA m6A demethylases ALKBH10B and ALKBH9B (homologs of the human m6A demethylase ALKBH5) affect floral transition [7] and viral infection [30]. Yu et al. (2021) improved crop yield by introducing the human m6A demethylase FTO to manipulate plant m6A levels [31]. PMEs system can specifically modify m6A level of targeted mRNAs, and therefore contributes to mining more m6A sites for high crop yield.

Sequences of the crRNA might be highly specific and critical for Cas13a activity [10, 19]. crRNA transcribed by RNA polymerase III promoters possess 3’ terminal poly U sequences, immediately next to protospacer sequences involved in RNA recognition [32]. The double ribozyme system was used precisely processes the gRNAs for Cas9 or CPF1 [15, 32]. By comparing results of PEMs with or without the double ribozyme system, it proves the relatively high efficiency of the double ribozyme system in PMEs (Fig. 3A-H). These findings have also laid the foundation for future usage of RNA polymerase II promoters. RNA polymerase II promoters provide better flexibility in operating temporal and spatial expressions of genes in vivo, and they can bypass short internal termination sites to produce long transcripts compared to RNA polymerase III promoters [33].

A further improvement of this system could be achieved by integrating nuclear export signal (NES), which changes the subcellular location of Cas13a-ALKBH fusion protein and enhances the editing efficiency [11, 12]. Meanwhile, the use of full-length ALKBH5 or a more active RNA targeting enzyme such as Cas13b in plants will also greatly improve the efficiency of demethylation [11, 12]. The PMEs system described here hold great promise to change the game play for future RNA regulation research.

## Conclusion

In summary, we developed a plant m6A editors (PMEs) using dLwaCas13a (from *L. wadei*) and human m6A demethylase ALKBH5 catalytic domain. We found that PMEs specifically demethylates m6A of targeted mRNAs to increase mRNAs stability. In addition, using the double ribozyme system could further improve the RNA editing efficiency of PMEs. The lack of effective tool makes it difficult to study the effect of specific RNA methylation and thus this tool has made a significant advance in the fields of RNA methylation.

## Methods

### Plant material and transformation

Arabidopsis (*Arabidopsis thaliana*) ecotype Columbia (Col-0) used in this study was kindly provided by Dr. Shenxiu Du (South China Agricultural University). The constructs were introduced into *A. tumefaciens* strain EHA105, and then transformed into Arabidopsis (Col-0) by the floral dip method. The seeds were collected from the T_0_ plants, screened on 1/2MS plates containing 25 mg/L hygromycin, and transplanted to soil. T_1_ plants were confirmed by PCR analysis of genomic DNA. DNA extraction was performed from young leaves of T_1_ plants using a CTAB protocol. PME-DEC-F and NOS-DEC-R designed according to the sequence of PME-MCS were used for detecting positive transgenic plants (Additional file 9). Plants were grown in pots of soil in controlled conditions at 22 ℃, under long day (16 h light/8 h dark). The homozygous lines were selected by examining the kanamycin resistance of T_3_ seedlings. The number of rosette leaves were counted at flowering time.

### Vector construction

The Arabidopsis codon optimized dLwaCas13a-msfGFP-XTEN-ALKBH^66–292^ was synthesized by GENEWIZ. This fragment together with CMV promoter was inserted into *EcoR*I-linearized pYL1300UaUf vector and formed PME-MCS using home-made Gibson Assembly mix [34, 35]. For PME-WS and PME-FSS, each crRNA expression cassette is composed of three parts, contain a snRNA promoter (AtU6), direct repeat (DR), and target sequence. crRNA expressing cassettes were assembled by single step overlap PCR according to previous method [36]. Briefly, the first round of PCR (20 µL) used four primers, the universal U-F and gRNA-R (0.2 mM each), and two target sequence-containing chimeric primers DR-R and T#-F (0.1 mM each), 0.2 U of Phanta Max Super-Fidelity DNA Polymerase (Vazyme), and pYLgRNA-AtU6-29 plasmid (20 ng each) as templates, for 25 cycles (94 °C, 10 s; 58 °C, 10 s; 72 °C, 15 s). The second round of PCRs (50 µL) were performed by using 0.4 µL of the first PCR products as templates, and 0.2 mM homologous sequences-containing chimeric primer pairs U-GA-# and Pts-GA-# (0.2 mM each). PME-MCS vector (100 ng) linearized with *Asc*I was mixed with purified PCR products of crRNA expression cassettes (15 ~ 20 ng each), and the mixture was adjusted to 5 µL, and then mixed with 5 µL of home-made Gibson Assembly mix. After incubation at 50 °C for 30 min, the product (1 µL) was transformed into commercial *E. coli* competent cells. The constructs PME-WS and PME-FSS were confirmed by PCR and DNA sequencing. For PME-WS-H and PME-FSS-H, crRNA expression cassettes, containing a snRNA promoter (AtU6) and four crRNAs flanked with double ribozyme system, were synthesized by GENEWIZ. Synthesized crRNA expression cassettes (15 ~ 20 ng) were inserted into PME-MCS vector (100 ng) linearized with *Asc*I using home-made Gibson Assembly mix [20]. The constructs PME-WS-H and PME-FSS-H were confirmed by PCR and DNA sequencing. Primers used in vector construct were listed in Additional file 9.

### Quantitative reverse transcription PCR (qPCR) analysis

For expression analysis, total RNA from Arabidopsis shoot apices (for expressional analysis of *WUS*, *STM*, *FT*, *SPL3* and *SPL9*) and leaves (for expressional analysis of five potential orthologs of human ALKBH5) were isolated using TRIzol reagent (Thermo, USA). Total RNA was used to synthesize cDNA from each sample using M-MLV Reverse Transcriptase (Promega, USA) according to the manufacturer’s instructions. Specific primers for qPCR were designed according to the gene CDS sequence and listed in Additional file 9. The qPCR was conducted using ChamQ SYBR qPCR Master Mix (Vazyme, China) with three biological repeats. *TUB2* (*At5g62690*) was used as an internal control to normalize target gene expression.

### m6A level analysis of target mRNAs

SELECT-qPCR is an efficient method for m6A level detection of target mRNAs. SELECT-qPCR was performed according to the method described previously [37]. Total RNAs (1500 ng) was mixed with 40 nM up primer, 40 nM down primer and 5 µM dNTP in 17 µL 1× CutSmart buffer (NEB, USA). The RNAs and primers were incubated as following: 90 ºC for 1 min, 80 ºC for 1 min, 70 ºC for 1 min, 60 ºC for 1 min, 50 ºC for 1 min and 40 ºC for 6 min. RNAs and primers mixture were incubated with 3 µL mixture of 0.01 U Bst 2.0 DNA polymerase (NEB, USA), 0.5 U SplintR ligase (NEB, USA) and 10 nM ATP (NEB, USA), at 40 ºC for 20 min, and then denatured at 80 ºC for 20 min. 2 µL of the final reaction mixture was used for SELECT -qPCR reaction. The PCR reaction cycle program was as follows: 95 ºC, 5 min; 95 ºC, 10 s then 60 ºC, 35 s for 40 cycles; 95 ºC, 15 s; 60 ºC, 1 min; 95 ºC, 15 s; 4 ºC, hold. Primers for SELECT-qPCR are listed in Additional file 8. All assays were performed with three independent experiments.

### Electronic supplementary material

Below is the link to the electronic supplementary material.

Additional file 1. Sequence of dlwCas13a-msfGFP-ALKBH cassette. Additional file 2. Sequence of crRNA expression cassette for PME-WS-H (Upper) and PME-FSS-H (Lower). Additional file 3. Secondary structure of crRNAs predicted by RNAfold. Additional file 4. PCR identification of T_1_ transgenic plants. Additional file 5. qPCR analysis of the expression of dCas13a-ALKBH in T_3_ transgenic seedlings. Additional file 6. qPCR analysis of the expression of five potential orthologs of human ALKBH5 in T_3_ transgenic plants leaves. Additional file 7. Real-time fluorescence amplification curves and bar plot of the threshold cycle (C_T_) of SELECT-qPCR. Additional file 8. Phenotypes of T_3_ transgenic seedlings. Additional file 9. Primers used in this study.

## Data Availability

The authors are pleased to share analyzed/raw data and plant materials upon reasonable request.
