# Supplementary material for: Targeted mRNA demethylation in Arabidopsis using plant m6A editor
Source: Plant Methods. 2023 Aug 9;19:81. doi: 10.1186/s13007-023-01053-7 (PMC10413771; doi:10.1186/s13007-023-01053-7)
Supplement: Supplementary file 1 — Additional file 1. Sequence of dlwCas13a-msfGFP-ALKBH cassette. Additional file 2. Sequence of crRNA expression cassette for PME-WS-H (Upper) and PME-FSS-H (Lower). Additional file 3. Secondary structure of crRNAs predicted by RNAfold. Additional file 4. PCR identification of T1 transgenic plants. Additional file 5. qPCR analysis of the expression of dCas13a-ALKBH in T3 transgenic seedlings. Additional file 6. qPCR analysis of the expression of five potential orthologs of human ALKBH5 in T3 transgenic plants leaves. Additional file 7. Real-time fluorescence amplification curves and bar plot of the threshold cycle (CT) of SELECT-qPCR. Additional file 8. Phenotypes of T3 transgenic seedlings. Additional file 9. Primers used in this study. [file 13007_2023_1053_MOESM1_ESM.docx]

ATGCCTAAGAAGAAGCGGAAGGTTGGTATTCACGGGGTGCCTGCGGCTTACCCCTACGACGTGCCCGACTACGCATACCCATACGACGTGCCGGACTACGCCTACCCGTACGACGTGCCCGACTACGCAATGAAGGTGACAAAGGTGGACGGTATCTCACACAAGAAGTACATCGAGGAGGGTAAGTTGGTGAAGTCAACATCAGAGGAGAACCGGACATCAGAGAGGTTGTCAGAGTTGTTGTCAATCCGGTTGGACATCTACATCAAGAACCCAGACAACGCATCAGAGGAGGAGAACCGGATCCGGCGCGAGAACTTGAAGAAGTTCTTCTCAAACAAGGTGTTGCACTTGAAGGACTCAGTGTTGTACTTGAAGAACCGGAAGGAGAAGAACGCAGTGCAGGACAAGAACTACTCAGAGGAGGACATCTCAGAGTACGACTTGAAGAACAAGAACTCATTCTCAGTGTTGAAGAAGATCTTGTTGAACGAGGACGTGAACTCAGAGGAGTTGGAGATCTTCCGGAAGGACGTGGAGGCAAAGTTGAACAAGATCAACTCATTGAAGTACTCATTCGAGGAGAACAAGGCAAACTACCAGAAGATCAACGAGAACAACGTGGAGAAGGTGGGTGGTAAGTCAAAGCGCAACATCATCTACGACTACTACCGGGAGTCAGCAAAGAGGAACGACTACATCAACAACGTGCAGGAGGCATTCGACAAGTTGTACAAGAAGGAGGACATCGAGAAGTTGTTCTTCTTGATCGAGAACTCAAAGAAGCACGAGAAGTACAAGATCCGGGAGTACTACCACAAGATCATCGGTAGGAAGAACGACAAGGAGAACTTCGCAAAGATCATCTACGAGGAGATCCAGAACGTGAACAACATCAAGGAGTTGATCGAGAAGATCCCAGACATGTCAGAGTTGAAGAAGTCACAGGTGTTCTACAAGTACTACTTGGACAAGGAGGAGTTGAACGACAAGAACATCAAGTACGCATTCTGCCACTTCGTGGAGATCGAGATGTCACAGTTGTTGAAGAACTACGTGTACAAGAGGTTGTCAAACATCTCAAACGACAAGATCAAGAGGATCTTCGAGTACCAGAACTTGAAGAAGTTGATCGAGAACAAGTTGTTGAACAAGTTGGACACATACGTGAGGAACTGCGGTAAGTACAACTACTACTTGCAGGTGGGTGAGATCGCAACATCAGACTTCATCGCAAGGAACCGGCAGAACGAGGCATTCTTGCGGAACATCATCGGTGTGTCATCAGTGGCATACTTCTCATTGAGGAACATCTTGGAGACAGAGAACGAGAACGGTATCACGGGGAGGATGAGGGGTAAGACAGTGAAGAACAACAAGGGTGAGGAGAAGTACGTGTCGGGGGAGGTGGACAAGATCTACAACGAGAACAAGCAGAACGAGGTGAAGGAGAACTTGAAGATGTTCTACTCATACGACTTCAACATGGACAACAAGAACGAGATCGAGGACTTCTTCGCAAACATCGACGAGGCAATCTCATCAATCGCACACGGTATCGTGCACTTCAACTTGGAGTTGGAGGGTAAGGACATCTTCGCATTCAAGAACATCGCACCATCAGAGATCTCAAAGAAGATGTTCCAGAACGAGATCAACGAGAAGAAGTTGAAGTTGAAGATCTTCAAGCAGTTGAACTCAGCAAACGTGTTCAACTACTACGAGAAGGACGTGATCATCAAGTACTTGAAGAACACAAAGTTCAACTTCGTGAACAAGAACATCCCATTCGTGCCATCATTCACAAAGTTGTACAACAAGATCGAGGACTTGAGGAACACATTGAAGTTCTTCTGGTCAGTGCCAAAGGACAAGGAGGAGAAGGACGCACAGATCTACTTGTTGAAGAACATCTACTACGGTGAGTTCTTGAACAAGTTCGTGAAGAACTCAAAGGTGTTCTTCAAGATCACAAACGAGGTGATCAAGATCAACAAGCAGAGGAACCAGAAGACGGGGCACTACAAGTACCAGAAGTTCGAGAACATCGAGAAGACAGTGCCAGTGGAGTACTTGGCAATCATCCAGTCAAGGGAGATGATCAACAACCAGGACAAGGAGGAGAAGAACACATACATCGACTTCATCCAGCAGATCTTCTTGAAGGGTTTCATCGACTACTTGAACAAGAACAACTTGAAGTACATCGAGTCAAACAACAACAACGACAACAACGACATCTTCTCAAAGATCAAGATCAAGAAGGACAACAAGGAGAAGTACGACAAGATCTTGAAGAACTACGAGAAGCACAACCGGAACAAGGAGATCCCACACGAGATCAACGAGTTCGTGAGGGAGATCAAGTTGGGTAAGATCTTGAAGTACACAGAGAACTTGAACATGTTCTACTTGATCTTGAAGTTGTTGAACCACAAGGAGTTGACAAACTTGAAGGGTTCATTGGAGAAGTACCAGTCAGCAAACAAGGAGGAGACATTCTCAGACGAGTTGGAGTTGATCAACTTGTTGAACTTGGACAACAACCGGGTGACAGAGGACTTCGAGTTGGAGGCAAACGAGATCGGTAAGTTCTTGGACTTCAACGAGAACAAGATCAAGGACCGCAAGGAGTTGAAGAAGTTCGACACAAACAAGATCTACTTCGACGGTGAGAACATCATCAAGCACCGGGCATTCTACAACATCAAGAAGTACGGTATGTTGAACTTGTTGGAGAAGATCGCAGACAAGGCAAAGTACAAGATCTCATTGAAGGAGTTGAAGGAGTACTCAAACAAGAAGAACGAGATCGAGAAGAACTACACAATGCAGCAGAACTTGCACCGGAAGTACGCAAGGCCAAAGAAGGACGAGAAGTTCAACGACGAGGACTACAAGGAGTACGAGAAGGCAATCGGTAACATCCAGAAGTACACACACTTGAAGAACAAGGTGGAGTTCAACGAGTTGAACTTGTTGCAGGGTTTGTTGTTGAAGATCTTGCACCGGTTGGTGGGTTACACATCAATCTGGGAGAGGGACTTGAGGTTCCGGTTGAAGGGTGAGTTCCCAGAGAACCACTACATCGAGGAGATCTTCAACTTCGACAACTCAAAGAACGTGAAGTACAAGTCGGGGCAGATCGTGGAGAAGTACATCAACTTCTACAAGGAGTTGTACAAGGACAACGTGGAGAAGAGGTCAATCTACTCAGACAAGAAGGTGAAGAAGTTGAAGCAGGAGAAGAAGGACTTGTACATCGCAAACTACATCGCACACTTCAACTACATCCCACACGCAGAGATCTCATTGTTGGAGGTGTTGGAGAACTTGAGGAAGTTGTTGTCATACGACCGGAAGTTGAAGAACGCAATCATGAAGTCAATCGTGGACATCTTGAAGGAGTACGGTTTCGTGGCAACATTCAAGATCGGTGCAGACAAGAAGATCGAGATCCAGACATTGGAGTCAGAGAAGATCGTGCACTTGAAGAACTTGAAGAAGAAGAAGTTGATGACAGACCGGAACTCAGAGGAGTTGTGCGAGTTGGTGAAGGTGATGTTCGAGTACAAGGCATTGGAGGGAGGTGGAGGTTCTGGAGGTGGTGGTTCTGGTGGAGGAGGATCAGTTTCTAAGGGTGAGGAGCTTTTCACTGGAGTGGTGCCTATCTTGGTTGAACTTGACGGAGATGTGAACGGACACAAGTTCTCAGTTAGGGGAGAAGGTGAAGGTGACGCTACAAACGGTAAGTTGACACTTAAGTTCATCTGTACTACTGGAAAGTTGCCAGTGCCTTGGCCTACTTTGGTGACTACACTTACTTACGGTGTGCAGTGTTTCTCTAGGTACCCAGATCACATGAAACAGCACGACTTTTTTAAATCAGCAATGCCAGAGGGATACGTGCAAGAAAGGACTATCTCTTTCAAGGACGACGGTACTTACAAAACAAGAGCTGAGGTTAAGTTCGAGGGTGACACTTTGGTGAATAGAATTGAGTTGAAAGGAATCGATTTTAAGGAAGATGGTAACATTCTTGGTCACAAGCTTGAGTACAACTTTAACTCACACAATGTTTATATTACAGCAGACAAACAAAAGAACGGTATCAAAGCTAACTTTAAGATTAGGCATAACGTTGAGGACGGATCAGTGCAGCTTGCAGATCACTATCAGCAGAATACTCCTATTGGAGACGGTCCAGTGCTTTTGCCAGATAACCACTACTTGTCTACTCAATCTAAATTGTCTAAGGATCCTAATGAGAAAAGGGACCATATGGTGCTTTTGGAGTTCGTGACTGCTGCTGGAATCACATTGGGTATGGACGAGTTGTATAAGGGTTCAGAGGGAGCTAAGTCCGGCAGCGAGACGCCAGGCACCTCCGAGAGCGCTACGCCTGAACCAGAGAGGTCAGACTACGAGGAGCAGCAGTTGCAGAAGGAGGAGGAGGCAAGGAAGGTGAAGTCGGGGATCCGGCAGATGAGGTTGTTCTCACAGGACGAGTGCGCAAAGATCGAGGCAAGGATCGACGAGGTGGTGTCAAGGGCAGAGAAGGGTTTGTACAACGAGCACACAGTGGACCGGGCACCATTGAGGAACAAGTACTTCTTCGGTGAGGGTTACACATACGGTGCACAGTTGCAGAAGAGGGGTCCGGGGCAGGAGAGGTTGTACCCACCGGGGGACGTGGACGAGATCCCAGAGTGGGTGCACCAGTTGGTGATCCAGAAGTTGGTGGAGCACCGGGTGATCCCAGAGGGTTTCGTGAACTCAGCAGTGATCAACGACTACCAGCCGGGGGGTTGCATCGTGTCACACGTGGACCCAATCCACATCTTCGAGAGGCCAATCGTGTCAGTGTCATTCTTCTCAGACTCAGCATTGTGCTTCGGTTGCAAGTTCCAGTTCAAGCCAATCCGGGTGTCAGAGCCAGTGTTGTCATTGCCAGTGAGGAGGGGTTCAGTGACAGTGTTGTCGGGGTACGCAGCAGACGAGATCACACACTGCATCCGGCCACAGGACATCAAGGAGAGGAGGGCAGTGATCATCTTGAGGAAGACAAGGTTGGACGCACCAAGGTTGAAGCGTCCTGCTGCCACCAAAAAGGCCGGACAGGCTAAGAAAAAGAAGTAG

**Additional file 1 Sequence of dlwCas13a-msfGFP-ALKBH cassette**. Sequences of dlwCas13a, msfGFP and ALKBH catalytic domain are marked in red, green, and blue, respectively.

AGGCTGTCTCGTCTCGTCTCACTAGTATGGAATCGGCAGCAAAGGAAAATATCAGAGATCTCTTACAGTTAGTTTCGTTCTTAATCCAAACTACTGCAGCCTGACAGACAAATGAGGATGCAAACAATTTTAAAGTTTATCTAACGCTAGCTGTTTTGTTTCTTCTCTCTGGTGCACCAACGACGGCGTTTTCTCAATCATAAAGAGGCTTGTTTTACTTAAGGCCAATAATGTTGATGGATCGAAAGAAGAGGGCTTTTAATAAACGAGCCCGTTTAAGCTGTAAACGATGTCAAAAACATCCCACATCGTTCAGTTGAAAATAGTAGCTCTGTTTATATATTGGTAGAGTCGACTAAGAGATTGAAATTACTGATGAGTCCGTGAGGACGAAACGAGTAAGCTCGTCGATTTAGACTACCCCAAAAACGAAGGGGACTAAAACAAGGGTAAGGCCATTTGTAGTGACGGCTGGCCGGCATGGTCCCAGCCTCCTCGCTGGCGCCGGCTGGGCAACATGCTTCGGCATGGCGAATGGGACGAGCTTGGAGTGGATGGGCTGGGAGTTCGTAGACGGAAACAAACGCAGAATCCAAGCGTGGAATCGGCAGCAAAGGAAATTACTGATGAGTCCGTGAGGACGAAACGAGTAAGCTCGTCGATTTAGACTACCCCAAAAACGAAGGGGACTAAAACGGAAAGGATTGCCCAAGACATTATCCATGGCCGGCATGGTCCCAGCCTCCTCGCTGGCGCCGGCTGGGCAACATGCTTCGGCATGGCGAATGGGACGAGCTTGGAGTGGATGGGCACTGAAGGTCCTCAATCGCACTGGAAACATCAAGGTCGGTGGAATCGGCAGCAAAGGAAATTACTGATGAGTCCGTGAGGACGAAACGAGTAAGCTCGTCGATTTAGACTACCCCAAAAACGAAGGGGACTAAAACCCATAGACATGGCTACTCATGTAGCCATGGCCGGCATGGTCCCAGCCTCCTCGCTGGCGCCGGCTGGGCAACATGCTTCGGCATGGCGAATGGGACGAGCTTGGAGTGGATGGCTGACCTCCTGCCAGCAATAGTAAGACAACACGCAAAGTCGTGGAATCGGCAGCAAAGGAAATTACTGATGAGTCCGTGAGGACGAAACGAGTAAGCTCGTCGATTTAGACTACCCCAAAAACGAAGGGGACTAAAACGCTTGATGATGATGATGCTGATGCTGTGGGCCGGCATGGTCCCAGCCTCCTCGCTGGCGCCGGCTGGGCAACATGCTTCGGCATGGCGAATGGGACGAGCTTGGAGTGGATGGTTTTTTTCAAGAGCTTGGAGTGGATGGATACGCGTCCAGGATACATAGATTACCACAACTC

AGGCTGTCTCGTCTCGTCTCACTAGTATGGAATCGGCAGCAAAGGAAAATATCAGAGATCTCTTACAGTTAGTTTCGTTCTTAATCCAAACTACTGCAGCCTGACAGACAAATGAGGATGCAAACAATTTTAAAGTTTATCTAACGCTAGCTGTTTTGTTTCTTCTCTCTGGTGCACCAACGACGGCGTTTTCTCAATCATAAAGAGGCTTGTTTTACTTAAGGCCAATAATGTTGATGGATCGAAAGAAGAGGGCTTTTAATAAACGAGCCCGTTTAAGCTGTAAACGATGTCAAAAACATCCCACATCGTTCAGTTGAAAATAGTAGCTCTGTTTATATATTGGTAGAGTCGACTAAGAGATTGAAATTACTGATGAGTCCGTGAGGACGAAACGAGTAAGCTCGTCGATTTAGACTACCCCAAAAACGAAGGGGACTAAAACGAAGGCCGAGATTGTAGATCTCAGCAAAGGCCGGCATGGTCCCAGCCTCCTCGCTGGCGCCGGCTGGGCAACATGCTTCGGCATGGCGAATGGGACGAGCTTGGAGTGGATGGGCTGGGAGTTCGTAGACGGAAACAAACGCAGAATCCAAGCGTGGAATCGGCAGCAAAGGAAATTACTGATGAGTCCGTGAGGACGAAACGAGTAAGCTCGTCGATTTAGACTACCCCAAAAACGAAGGGGACTAAAACGAGAGTAAGCAAAGCCTGTTTCATTAACGGCCGGCATGGTCCCAGCCTCCTCGCTGGCGCCGGCTGGGCAACATGCTTCGGCATGGCGAATGGGACGAGCTTGGAGTGGATGGGCACTGAAGGTCCTCAATCGCACTGGAAACATCAAGGTCGGTGGAATCGGCAGCAAAGGAAATTACTGATGAGTCCGTGAGGACGAAACGAGTAAGCTCGTCGATTTAGACTACCCCAAAAACGAAGGGGACTAAAACCTCTGATGCAAAGACAAGTCAGAGAGACGGCCGGCATGGTCCCAGCCTCCTCGCTGGCGCCGGCTGGGCAACATGCTTCGGCATGGCGAATGGGACGAGCTTGGAGTGGATGGCTGACCTCCTGCCAGCAATAGTAAGACAACACGCAAAGTCGTGGAATCGGCAGCAAAGGAAATTACTGATGAGTCCGTGAGGACGAAACGAGTAAGCTCGTCGATTTAGACTACCCCAAAAACGAAGGGGACTAAAACCAACAACTCTGCTTACTATAAGAGGGTCGGCCGGCATGGTCCCAGCCTCCTCGCTGGCGCCGGCTGGGCAACATGCTTCGGCATGGCGAATGGGACGAGCTTGGAGTGGATGGTTTTTTTCAAGAGCTTGGAGTGGATGGATACGCGTCCAGGATACATAGATTACCACAACTC

**Additional file 2 Sequence of crRNA expression cassette for PME-WS-H (Upper) and PME-FSS-H (Lower)**. Sequences of AtU6 promoter, double ribozyme system and direct repeat are marked in red, green, and blue, respectively.

**
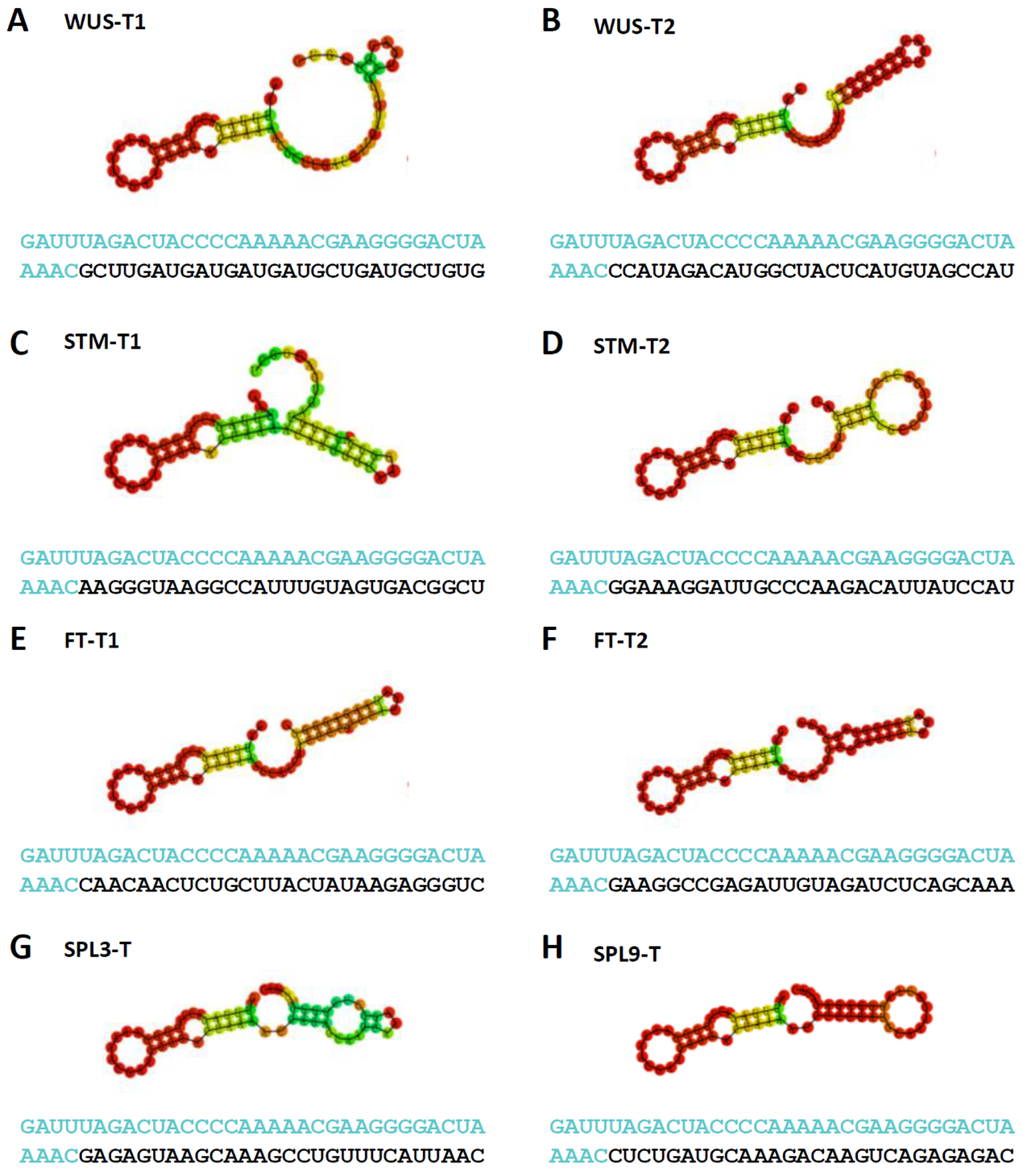
**

**Additional file 3 Secondary structure of crRNAs predicted by RNAfold.**


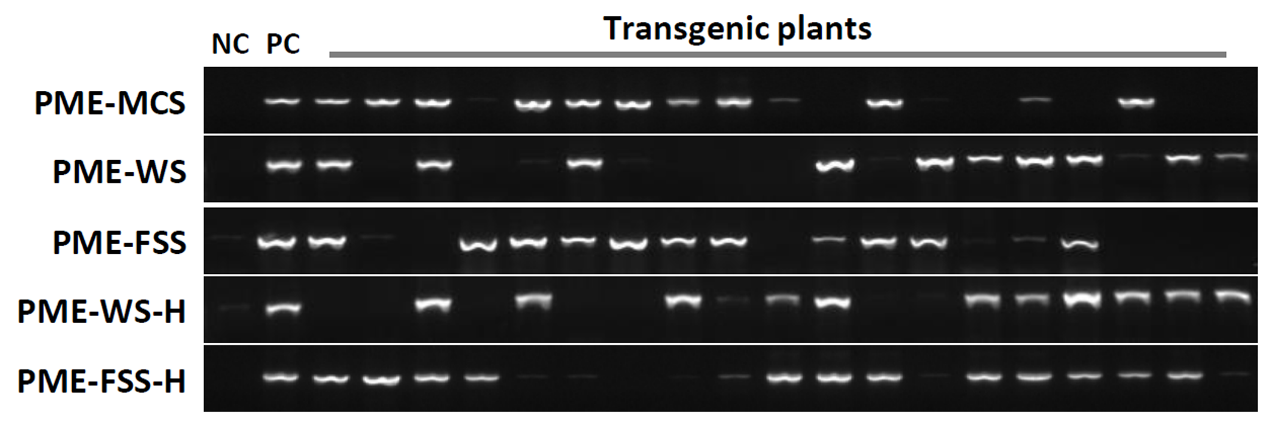


**Additional file 4 PCR identification of T_1_ transgenic plants.** NC, negative control. PC, positive control.


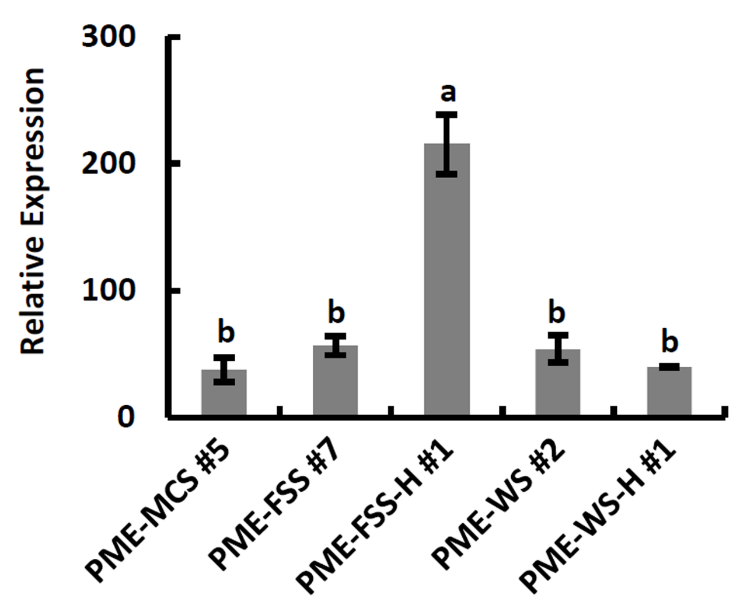


**Additional file 5 qPCR analysis of the expression of dCas13a-ALKBH in T_3_ transgenic seedlings.** *TUB2* was used as internal control. Error bars show SD (n = 3). Different letters at the top of each column indicate a significant difference at p < 0.05 determined by the Tukey test.


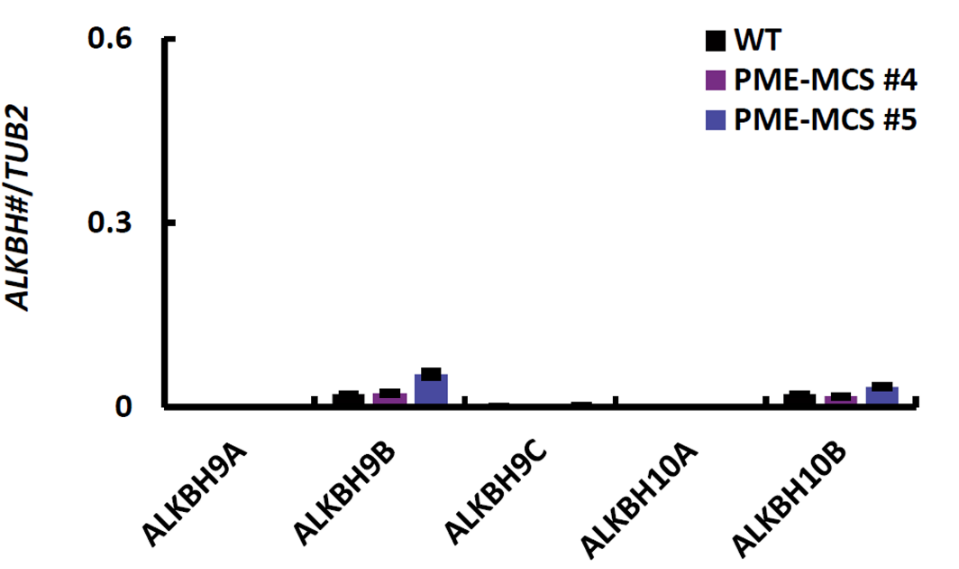


**Additional file 6 qPCR analysis of the expression of five potential orthologs of human ALKBH5 in T_3_ transgenic plants leaves.** *TUB2* was used as internal control. Error bars show SD (n = 3).

**
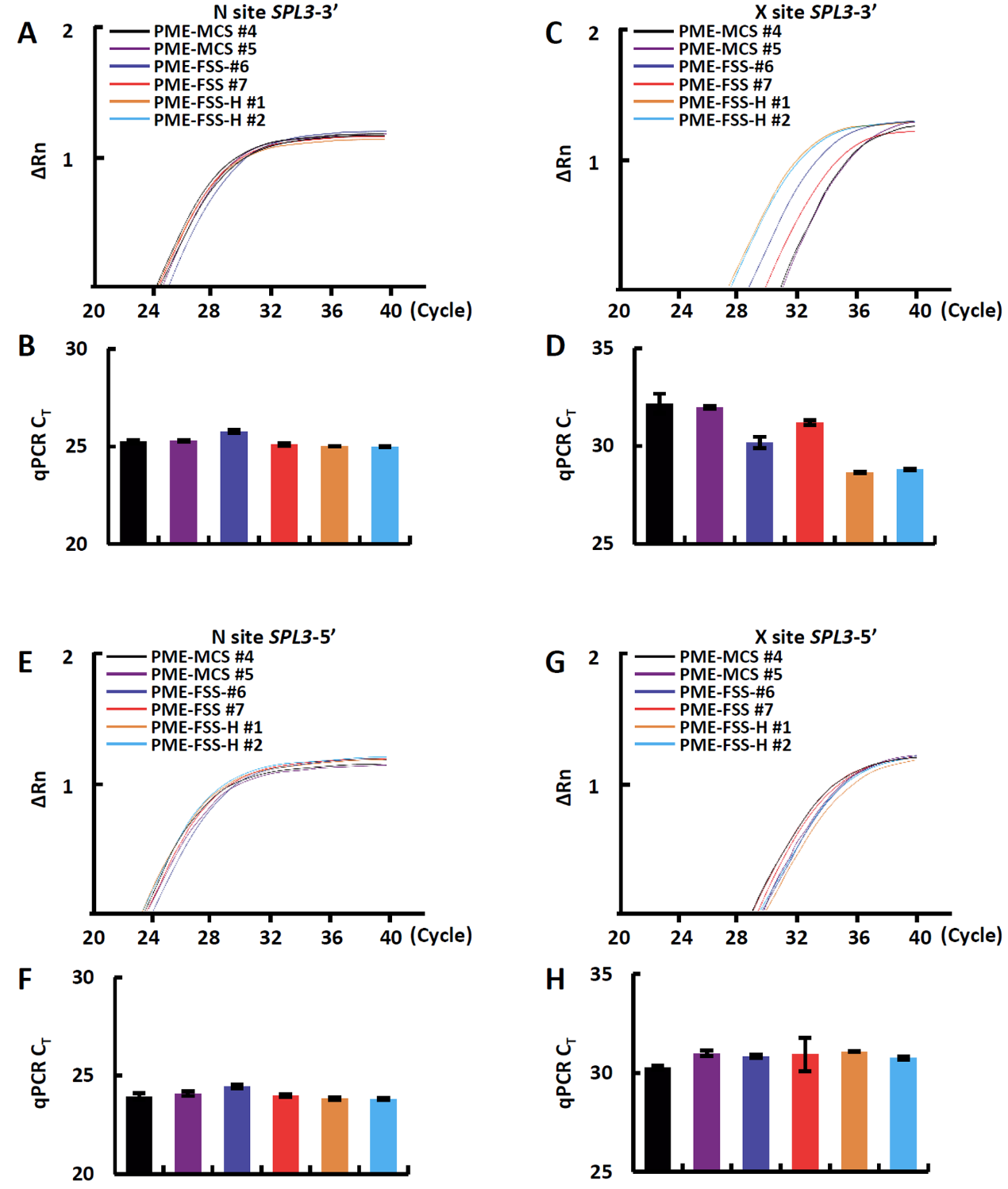
**

**Additional file 7 Real-time fluorescence amplification curves and bar plot of the threshold cycle (C_T_) of SELECT-qPCR.** N site was used as input control**.** Error bars show SD (n = 3).

**
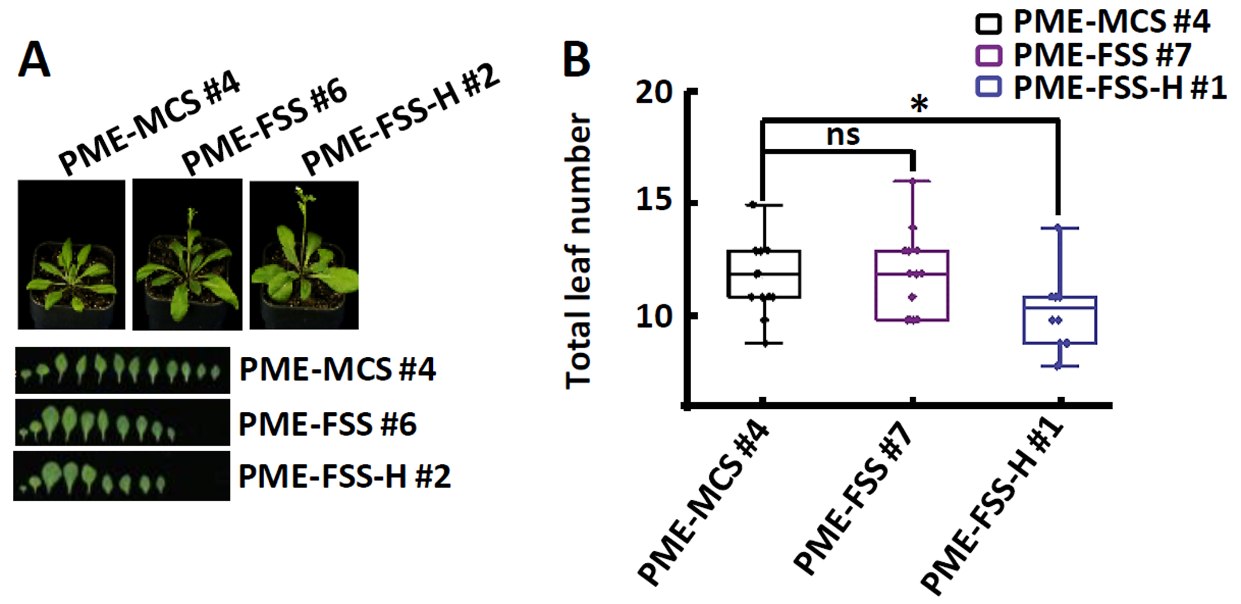
**

**Additional file 8 Phenotypes of T_3_ transgenic seedlings.** (A) Phenotypes of T_3_ transgenic seedlings carrying PME-MCS, PME-FSS, and PME-FSS-H, respectively. (B) The number of rosette leaves at the time of bolting in T_3_ transgenic plants carrying PME-MCS, PME-FSS, and PME-FSS-H grown in long days, respectively. Error bars show SD (n = 12). Asterisks indicate the significant differences between the two groups (Student’s t test, * p < 0.05; ** p < 0.01).

| Primer | Sequence (5’ to 3’) | Purpose |
| --- | --- | --- |
| U-F | CTCCGTTTTACCTGTGGAATCG | Vector construction |
| gR-R | CGGAGGAAAATTCCATCCAC |  |
| DR-R | GTTTTAGTCCCCTTCGTTTTTGGGGTAGTCTAAATCCAATCTCTTAGTCGACTCTACCA |  |
| WUS-T_1_-F | AAGGGGACTAAAACGCTTGATGATGATGATGCTGATGCTGTG TTTTTTTCAAGAGCTTG |  |
| WUS-T_2_-F | AAGGGGACTAAAACCCATAGACATGGCTACTCATGTAGCCAT TTTTTTTCAAGAGCTTGGAGTGGATG |  |
| STM-T_1_-F | AAGGGGACTAAAACAAGGGTAAGGCCATTTGTAGTGACGGCT TTTTTTTCAAGAGCTTGGAGTGGATG |  |
| STM-T_2_-F | AAGGGGACTAAAACGGAAAGGATTGCCCAAGACATTATCCAT TTTTTTTCAAGAGCTTGGAGTGGATG |  |
| FT-T_1_-F | AAGGGGACTAAAACCAACAACTCTGCTTACTATAAGAGGGTC TTTTTTTCAAGAGCTTGGAGTGGATG |  |
| FT-T_2_-F | AAGGGGACTAAAACGAAGGCCGAGATTGTAGATCTCAGCAAA TTTTTTTCAAGAGCTTGGAGTGGATG |  |
| SPL3-T-F | AAGGGGACTAAAACGAGAGTAAGCAAAGCCTGTTTCATTAAC TTTTTTTCAAGAGCTTGGAGTGGATG |  |
| SPL9-T-F | AAGGGGACTAAAACCTCTGATGCAAAGACAAGTCAGAGAGAC TTTTTTTCAAGAGCTTGGAGTGGATG |  |
| U-GA-1 | AGGCTGTCTCGTCTCGTCTCACTAGTATGGAATCGGCAGCAAAGG |  |
| Pts-GA-1 | CAGGGAGCGGATAACAATTTCACACAGGCACATCCACTCCAAGCTCTTG |  |
| U-GA-2 | GTGCCTGTGTGAAATTGTTATCCGCTCCCTGGAATCGGCAGCAAAGG |  |
| Pts-GA-2 | CCACGCATACGATTTAGGTGACACTATAGCGCATCCACTCCAAGCTCTTG |  |
| U-GA-3 | CGCTATAGTGTCACCTAAATCGTATGCGTGGTGGAATCGGCAGCAAAGG |  |
| Pts-GA-3 | GTCGCTAGTTATTGCTCAGCGGCCAAGCTCATCCACTCCAAGCTCTTG |  |
| U-GA-4 | GAGCTTGGCCGCTGAGCAATAACTAGCGACTGGAATCGGCAGCAAAGG |  |
| Pts-GA-4 | GAGTTGTGGTAATCTATGTATCCTGGACGCGTATCCATCCACTCCAAGCTCTTG |  |
| PME-DEC-F | CACATACGGTGCACAGTTGC | Detection of the transgene. |
| NOR-DEC-R | CCGATCTAGTAACATAGATGACACC |  |
| WUS-F | CAGAGACCTGCTAATTCCGTCA | qRT-PCR |
| WUS-R | CCACATTCAGTACCTGAGCTTGC |  |
| STM-F | ATCATGGCTCATCCTCACTACC |  |
| STM-R | GACATCCTGTTGGTCCCATAGA |  |
| FT-F | GCTACAACTGGAACAACCTTTGGC |  |
| FT-R | TCGCGAGTGTTGAAGTTCTGGC |  |
| SPL3-F | CTTAGCTGGACACAACGAGAGAAGGC |  |
| SPL3-R | GAGAAACAGACAGAGACACAGAGGA |  |
| SPL9-F | CAAGGTTCAGTTGGTGGAGGA |  |
| SPL9-R | TGAAGAAGCTCGCCATGTATTG |  |
| 13a-QF | AGAGGCCAATCGTGTCAGTGT |  |
| 13a-QR | TCCTCACTGGCAATGACAACACT |  |
| TUB2-F | ATCCGTGAAGAGTACCCAGAT |  |
| TUB2-R | AAGAACCATGCACTCATCAGC |  |
| qPCR-SELECT-F | ATGCAGCGACTCAGCCTCTG | SELECT-qPCR |
| qPCR-SELECT-R | TAGCCAGTACCGTAGTGCGTG |  |
| SPL9-X-UP | TAGCCAGTACCGTAGTGCGTGATCTGGCAACAATTGTCTGG |  |
| SPL9-X-DOWN | TTATTGATAGCAGATAACAGAATAAACAAAGCAGAGGCTGAGTCGCTGCAT |  |
| SPL9-N-UP | TAGCCAGTACCGTAGTGCGTGGGCAACAATTGTCTGGTTTATTGA |  |
| SPL9-N-DOWN | AGCAGATAACAGAATAAACAAAGAAGCCAGAGGCTGAGTCGCTGCAT |  |
| FT-X-UP | TAGCCAGTACCGTAGTGCGTGATATCAATTGGTTATAAAGGAAGAAGCCATCTAAAG |  |
| FT-X-DOWN | CTTCTTCCTCCGCAGCCACCAGAGGCTGAGTCGCTGCAT |  |
| FT-N-UP | TAGCCAGTACCGTAGTGCGTGTTATAAAGGAAGAAGCCATCTAAAGTCTTCTTCC |  |
| FT-N-DOWN | CCGCAGCCACTCTCCCTCTGCAGAGGCTGAGTCGCTGCAT |  |
| SPL3-3̓-X-UP | TAGCCAGTACCGTAGTGCGTGATTACACATAATTTCTTCAAGCATTTACTAAG |  |
| SPL3-3̓-X-DOWN | CTCAATGCATTTATTTAACCATGTCGCAGAGGCTGAGTCGCTGCAT |  |
| SPL3-3̓-N-UP | TAGCCAGTACCGTAGTGCGTGCAAGCATTTACTAAGTCTCAATGCA |  |
| SPL3-3̓-N-DOWN | TTATTTAACCATGTCGTAGGTTTAGCCAGAGGCTGAGTCGCTGCAT |  |
| SPL3-5̓-X-UP2 | TAGCCAGTACCGTAGTGCGTGCTTCTTCCTCTTCCTCACTCAG |  |
| SPL3-5̓-X-DOWN2 | TCTCGTAAGCTCCTCTTCCCAGAGGCTGAGTCGCTGCAT |  |
| SPL3-5̓-N-UP2 | TAGCCAGTACCGTAGTGCGTGACTCAGTTCTCGTAAGCTCC |  |
| SPL3-5̓-N-DOWN2 | CTTCCCTTCCGCTTTGCTTCAGAGGCTGAGTCGCTGCAT |  |

**Additional file 9 Primers used in this study.**
